# Monitoring Everyday Upper Extremity Function in Patients with Complex Regional Pain Syndrome: A Secondary, Retrospective Analysis from ncRNAPain

**DOI:** 10.1155/2024/9993438

**Published:** 2024-08-24

**Authors:** Gudrun-Karin Kindl, Ann-Kristin Reinhold, Fabiola Escolano-Lozano, Johannes Degenbeck, Frank Birklein, Heike L. Rittner, Karolin Teichmüller

**Affiliations:** ^1^ University Hospital Würzburg Department of Anaesthesiology Intensive Care, Emergency and Pain Medicine Centre for Interdisciplinary Pain Medicine, Würzburg, Germany; ^2^ University Hospital of Mainz Department of Neurology, Mainz, Germany

## Abstract

**Objective:**

Complex regional pain syndrome (CRPS) represents a rare complication following injury to a limb. The DASH questionnaire (disability of arm, shoulder, and hand) evaluates everyday arm function. We assessed the DASH and its subitems in comparison to patients with brachial plexus lesions or fracture controls, analysed it over time, and in relation to active range of motion (ROM), to determine patients' impairment and trajectory.

**Methods:**

The dataset included 193 patients with upper extremity CRPS from the noncoding RNA (ncRNA) Pain cohort, 36 fracture controls, and 12 patients with traumatic brachial plexus lesions. For the clinical and psychological characterisation, questionnaires and a goniometer for the measurement of ROM were utilized. Thirty-three patients were followed up after approximately 2.5 years of guideline treatment.

**Results:**

CRPS patients had a similar mean DASH of 54.7 (standard deviation (S.D.) ±21) as brachial plexus lesion patients (*M* = 51.4, S.D. ± 16.1) but different significantly from fracture controls (*M* = 21.2, S.D. ± 21.1). Pain and older age were predictors of the DASH. Activities requiring force or impact on the arm, shoulder, or hand were mostly affected in patients with CRPS. After 2.5 years of standard treatment, the mean DASH score fell to 41.3 (S.D. ± 25.2), weakness in leisure activities was recuperated, pain feelings were lessened, and ROM, e.g., wrist flexion, recovered by 36°. Two-thirds of patients improved in both the DASH and the ROM.

**Conclusions:**

CRPS is as disabling as a complete loss of arm function in brachial plexus lesions and exhibits only partial recovery. Developing QuickDASH versions for CRPS patients could reduce the load of questions in clinical studies. It would be prudent to consider the unexpected age dependency of the DASH in future studies. This trial is registered with DRKS00008964.

## 1. Introduction

Complex regional pain syndrome (CRPS) is a condition that develops subsequent to fractures or soft tissue injuries of the extremities. The incidence of this disease is 5.5 per 100,000 person-years in North America [1]. Women are affected 2-3 times more often and most patients are between 50 and 70 years old [2]. Clinicians diagnose the disease using official International Association for the Study of Pain (IASP) approved criteria: Patients suffer from excessive pain “disproportionate to the preceding trauma”; sensory alterations (hyperalgesia and allodynia); and autonomic changes such as edema, temperature differences, skin color changes, sweating, and motor and trophic changes [3]. Depending on the nerve lesion involved, CRPS is classified as type I or type II [4]. CRPS is often accompanied by a loss of motor function of the affected extremity, including force reduction, incomplete fist closure, not fully extendable fingers, or restrictions in fine motor skills [5]. These symptoms can lead to a long-term impact on work capability [6]. Nevertheless, there is a paucity of knowledge regarding the prevalence of disability in daily life and the manner in which impairments and abilities evolve over time [7, 8].

The DASH (disability of the arm, shoulder, and hand) questionnaire was developed and validated by the American Academy of Orthopedic Surgeons' outcome research committee in 1994 to evaluate pain and functional outcomes for disabilities of the upper extremity [9, 10]. The DASH has been designed to self-assess symptoms and functional status in populations with upper extremity musculoskeletal conditions. Most of its score reflects physical function in everyday life, while symptoms, e.g., pain or sleep, influence the calculated DASH score to a minor degree [9]. In 2003, the DASH was translated into German and proved reliable (*r*_*s*_ = 0.9) and valid (rho: 0.76) for German-speaking patients [10, 11]. The DASH has also been validated in several other countries and has been demonstrated to be a valid and reliable instrument for measuring standardized patient-centred outcomes in musculoskeletal impairments of the upper extremities [12, 13]. However, it is quite extensive and redundant. Therefore, Beaton and colleagues [14] reduced the original DASH (score ranges from 0 to 100% disability) to the 11-item QuickDASH (score ranges from 0 to 100% disability) with good psychometric properties [15]. Both the DASH and QuickDASH assess arm function in patients with any musculoskeletal disorder of the upper limb with higher scores indicating higher levels of disability [11, 12].

The DASH has rarely been utilized in the literature to describe arm function in CRPS: Savas et al. identified a DASH value twice as high compared to their control group (55 vs 26) [16]. Askin et al. observed an average initial DASH of 73–88 in their cohorts, which improved over time, from 37 to 52, following stellate ganglion ultrasound, similar medication, and transcutaneous electrical nerve stimulation [17]. However, it is unclear how CRPS differs from other diseases of the upper extremities, e.g., traumatic brachial plexus lesions or arm fractures that have healed without complications as a “negative” comparator. Brachial plexus lesions represent the most severe injuries of the upper extremity, frequently resulting from high-impact traumas such as motorcycle accidents [18]. Novak and colleagues [19] used, amongst others, the DASH to demonstrate that brachial plexus lesions lead to severe pain disability and functional impairment, rendering them suitable “positive” comparators. We aimed to compare pain, disability, and upper extremity function in CRPS patients at study enrolment and after 2.5 years of disease duration. This cohort was compared with patients with brachial plexus lesions and patients with a normal healing process following a fracture of the upper extremity. By contributing to a better understanding of patients' impairment and trajectory, implications for optimized treatment approaches can be derived.

Using a large cohort, we aimed to focus on self-reported everyday function rather than on pain in CRPS to estimate the severity of the disease. To this end, we seek to explore how disabled CRPS patients are comparing them to mildly affected fracture controls with normal arm fracture healing and severely impaired brachial plexus injury, and how DASH scores change during the disease. Second, we aimed to identify factors determining DASH including reported pain and measured joint mobility.

## 2. Materials and Methods

### 2.1. Study Design

We conducted a large study funded by the EU “ncRNAPain,” study protocol registered at the German Clinical Trial Register (https://drks.de/) (Registration no. DRKS00008964), and prospectively collected data from CRPS patients recruited from outpatient pain clinics in Würzburg and Mainz [3, 20]. In a secondary analysis, a retrospective cohort study was conducted. One of these groups provided data for a longitudinal follow-up analysis after ∼2.5 years. During this period, they received individualised treatment in accordance with the German CRPS treatment guideline, which included differential pharmacological treatment and occupational and physical therapy [21].

### 2.2. Sample Size

Following approval by the ethics committee (52/14_z), the data from four different cohorts were analysed collectively.

**Figure 1:**
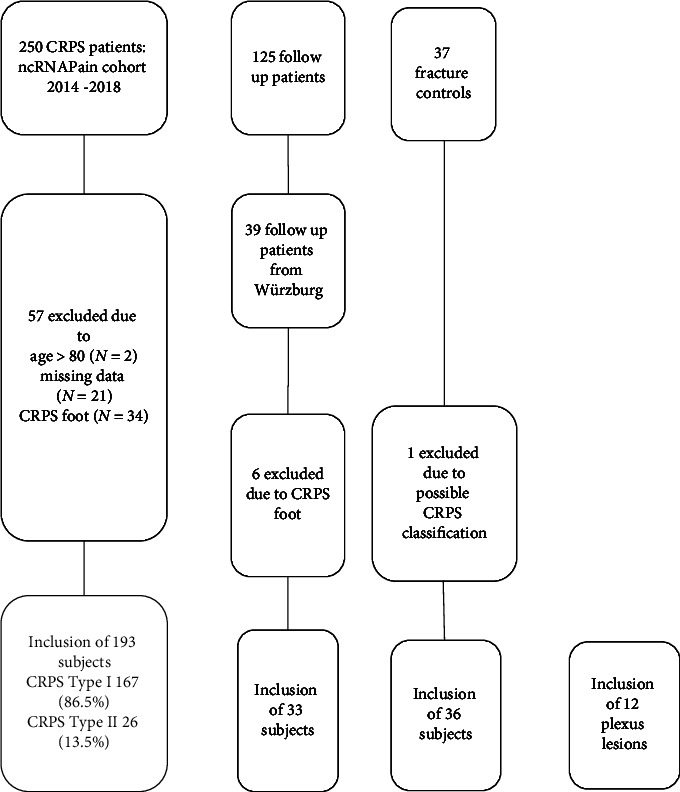
Flowchart of the analysis. CRPS = complex regional pain syndrome group, time point T0 study inclusion. Follow-up group after ø 2.5 years, time point T1; 36 fracture controls with normal trauma healing within one year after trauma; 12 plexus lesion patients with dorsal root avulsion after mainly motorcycle accidents.

The ncRNAPain CRPS cohort comprised 250 data entries, which were reviewed by the ncRNAPain study group. A total of 57 subjects (22.8%) were excluded due to the following reasons: (i) age < 18 and >80 years (*N* = 2), (ii) incomplete data (*N* = 21), and (iii) CRPS of the foot (*N* = 34). Finally, we included 193 CRPS patients; 86% with CRPS type I (Figure 1).

Among the 125 follow-up patients from all study centres, 39 subjects from Würzburg were examined after an average (ø) of 2.5 years and included in the study, with 82% exhibiting CRPS type I. During the follow-up period, patients received individualised treatment in accordance with the German CRPS treatment guideline [21, 22]. Six patients were excluded due to CRPS of the foot (Figure 1).

An important control group of patients was fracture controls who had experienced normal fracture healing. Further details regarding this subgroup can be found in [3]. There were 37 subjects in this group; one had to be excluded due to possible CRPS based on a retrospective chart review.

Twelve patients, mainly after motorcycle accidents, with dorsal root ganglion avulsion were examined [23] five months after the accident when they were scheduled for nerve transfer surgery. This injury resulted in a lesion of the brachial plexus, which significantly impaired the function of the affected arm.

### 2.3. Procedures

For all groups, study files (with supplementation by primary chart review) were used to extract the following parameters: gender, age, DASH score and its items, pain intensity using the numeric rating scale (NRS, 0–10, no pain to maximum pain), and neuropathic pain score inventory (NPSI) score. In addition, we collected data on height, weight, and BMI; von Korff score; CSS (CRPS severity score); comorbidities (depression and anxiety characteristics, measured with the BDI-II and STAI-T); pain duration; and range of motion of the wrist, digit D II, and digit D V. Plexus lesion patients were further described in a previous publication [23]. Data were collected at study inclusion and again in the follow-up cohort after 2.5 years.

### 2.4. Outcome Measurements

The DASH consists of three question modules: (1) the general module with 30 questions on function/symptoms and social roles. They include 21 physical function items (e.g., make a bed, recreational activities, gardening or yard work, and carrying a heavy object); six symptom items about pain, tingling, weakness, stiffness, and sleeping problems; and three social role items (e.g., confidence to perform tasks); (2) the optional high-performance sport or music module; and (3) the optional work module. In our investigation, we concentrated on the first module in the German version [7]. For each question, the subject rated the performance in the last week on a 5-point scale (1 = no difficulty to perform or no impact; 5 = unable to do or high impact). The raw score is the sum of all answers (maximum score: 150; minimum score: 30). The raw score is transformed into a zero to 100 scale (= total DASH). Zero is no disability and 100 maximum disability [10].

The Neuropathic Pain Symptom Inventory (NPSI) is a self-questionnaire, developed by Bouhassira et al. in 2003 to investigate the individual characteristics of neuropathic pain [24]. It consists of 13 points that query symptoms such as burning, stinging, or tingling. The rating scale ranges from 0 to 10, where 0 indicates no symptom and 10 is maximum symptom expression. Subscores for burning, pressing, paroxysmal, evoked pain, or paresthesia/dysesthesia can be calculated. The subscores can be aggregated and divided by 100 to yield a total score between 0 and 100, with 0 indicating no symptom and 100 representing the maximum possible symptom expression.

The severity of chronic pain was evaluated using the von Korff score, which measures both pain intensity and pain-related disability. The von Korff score enables the differentiation of patients with high pain scores who are not disabled from patients with comparable pain who are significantly disabled. It is divided into a 4 level categorical variable (grade 0: no pain; grade 1: low disability-low intensity; grade 2: low disability-high intensity; grade 3: high disability-moderately limiting; and grade 4: high disability-severely limiting [25, 26]).

Harden et al. developed the CRPS severity score (CSS) to identify the severity and liability of CRPS symptoms such as pain, oedema, and limited motor function. It comprises eight patient-reported symptoms and nine investigator-observed signs [27, 28]. The current symptoms are aggregated. The total CSS score ranges from 17 to 0, with higher values indicating a greater number of symptoms and a higher level of impairment.

To estimate depressive symptoms, patients answered the Beck Depression Inventory 2, utilizing the validated German version [29] (BDI-II, range 0–63; 0–13 indicative of minimal depression, 14–19 mild depression, 20–28 moderate depression, and 29–63 severe depression). Anxiety characteristics were measured by the State-Trait Anxiety Inventory, German Version [30] (trait anxiety subscale STAI-T, range 20–80). For the STAI-T, a value ≤ 39 was defined as normal [3]. In addition, we asked the study subjects if they had a history of anxiety or depression.

With the range of motion (ROM), we objectively measured the active movement of the joints with a goniometer while the patients' elbows rested on a table. There is a discrepancy in the definition of normal values among authors [31, 32]. In the present study, we focused on the motility of the wrist and the proximal interphalangeal joints of fingers D II and D V. As normal values for the wrist, we defined 60°–0°–70° starting with the extension, followed by the neutral position and the flexion. For D II, we set 0°-0°–110° and for D V 0°-0°–100° as normal. Deviating values were classified as pathological. ROM was measured at the first visit in the CRPS group and after an average of 2.5 years in the follow-ups to assess the change in ROM over time.

### 2.5. Statistical Analysis

Data were collected and listed in Excel 2016 and SPSS 28 tables. Using SPSS 28 for all statistical tests to analyse group differences, we performed Pearson chi-square test for nominal data (gender, depression, and anxiety), Mann–Whitney *U* test for not paired ordinal outcome measures, and Wilcoxon test for paired ordinal outcome measures and for metric data that were not normally distributed (DASH, ROM, von Korff, NPSI, CSS, BDI-II, STAI-T, age, BMI, height, pain intensity, and pain duration in years). Figures and tables were created with GraphPad Prism 9, Excel 2016, and Word 2016. The significance level was set at 5%, and multiple linear regression and Pearson's correlation were calculated, where data were normally distributed. Group differences in DASH, NRS, NPSI, and CSS were calculated using the Kruskal–Wallis test with post hoc Dunn–Bonferroni correction.

## 3. Results

### 3.1. Patient Cohorts

In the CRPS cohort and its subsequent follow-up, there was a preponderance of female participants, whereas in the plexus group, there were a greater proportion of male participants. The gender ratio was 1 : 1 for the fracture controls (Table 1). Age and BMI were similarly distributed in CRPS patients, follow-up, and fracture controls, with fracture controls being slightly younger and lighter in weight. The plexus lesion group was more than 10 years younger; BMI values were not available for this subgroup.

### 3.2. Everyday Functional Impairment in CRPS Patients Is as Pronounced as after Plexus Injury

**Figure 2:**
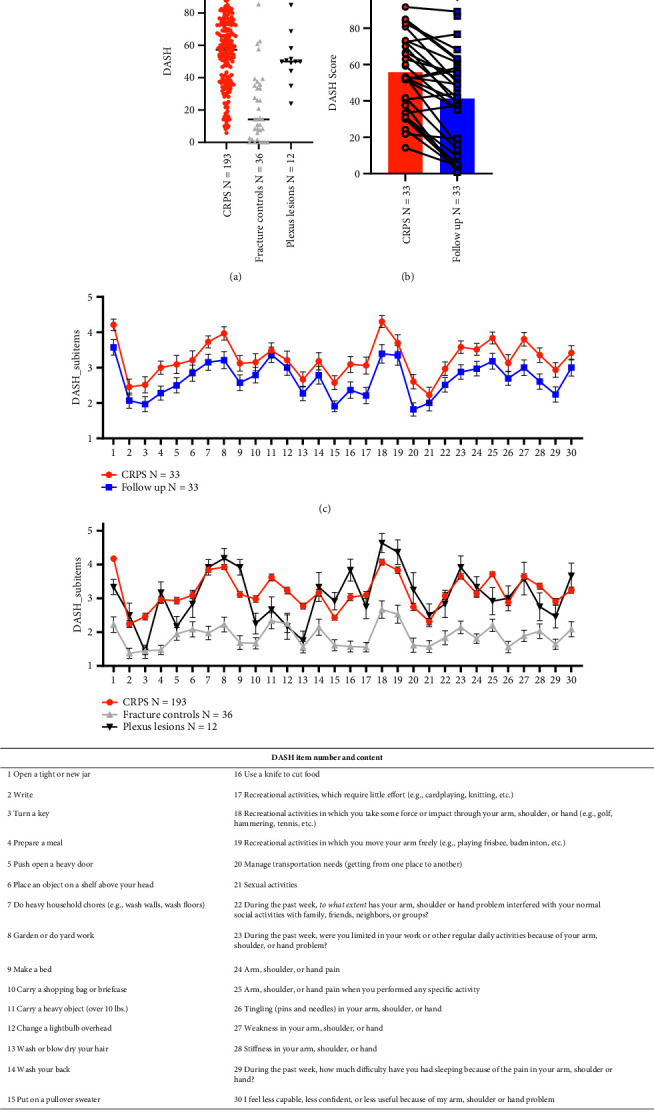
Severe functional disability of CRPS patients and its small longitudinal improvement measured by the DASH score. (a) DASH score in CRPS, fracture controls, and plexus lesion group (0–100; 0 = no impairment and 100 maximum impairment). (b) DASH score of CRPS patients at the time of inclusion and after 2.5 years in the follow-up. (c) Mean of DASH items of the CRPS group at the study inclusion and follow-up (1–5; 1 = low impact and 5 = very high impairment). (d) Mean of DASH items of patients with CRPS, fracture controls, and plexus lesions. Numbers and content of the individual questions. ^*∗∗∗*^*p* < 0.001. ^*∗∗∗∗*^*p* < 0.0001. Nonparametric Kruskal–Wallis and Wilcoxon tests, no normal distribution.

The mean DASH score was highest in the CRPS group (54.7, S.D. ± 21), even higher in CRPS type II (56.9, S.D. ± 20.5) vs. type I (54.3, S.D. ± 21.5), followed by the plexus lesion group. Both groups were not significantly different in DASH scores (Figure 2(a)). As expected, fracture controls had the lowest DASH values. After 2.5 years, physical impairment measured by DASH significantly improved, however, with clear restrictions in the arm function in everyday life (Figure 2(b)). The mean DASH score at follow-up was still 20 points higher than in fracture controls. During follow-up, patients were managed as per the CRPS guideline, which includes differential treatment of CRPS (pharmacological, occupational, and physical therapy) [21].

The subsequent analysis focused on the single items of the DASH to determine the impact of individual everyday activities in CRPS patients (Figure 2(c)). The most severe impairment, based on the mean value, occurred when performing recreational activities that involved force or impact through the arm, shoulder, or hand (e.g., golf, hammering, and tennis; question 18); opening a tight or new jar (question 1); and gardening (question 8) (mean disability: 4.2 ± 1.0). In contrast, CRPS patients exhibited the least difficulties with sexual activity (question 21), writing (question 2), and turning a key (question 3) (mean disability: 2.4 ± 1.3).

The strongest improvement at follow-up was observed for recreational activities requiring force or impact through the arm, shoulder, or hand (e.g., golf, hammering, and tennis; question 18). In addition, weakness in the arm (question 27) and activity-related pain (question 25) were noted (mean improvement 2.0, Figure 2(c)). Overall, approximately 80% of CRPS patients experienced an improvement of 20% on average in function as measured by the DASH score.

The distribution of the most challenging activities was comparable for plexus lesions (Figure 2(d); questions 18, 19, and 8, with 19 containing recreational activities in which you move your arm freely (e.g., playing frisbee and badminton)). The least difficult items were 13 (wash or blow dry your hair), 12 (change a lightbulb overhead), and 5 (push open a heavy door).

### 3.3. Pain Correlates with DASH

We recorded the minimum, maximum, and mean pain intensity on the day of the presentation or in the week preceding the presentation at the study centre for each group. In our analysis, we concentrated on mean pain intensity, as this construct of “average pain” is most closely aligned with the instructions for completing the DASH. CRPS and plexus lesion patients experienced the highest levels of pain across all three categories, while those fracture controls exhibited minimal discomfort compared to all other groups. Over time, all pain scores decreased significantly, with an average reduction of one point on the NRS scale. CSS and von Korff decreased as a sign of symptom relief, and NPSI increased slightly but not significantly (Table 1).

**Figure 3:**
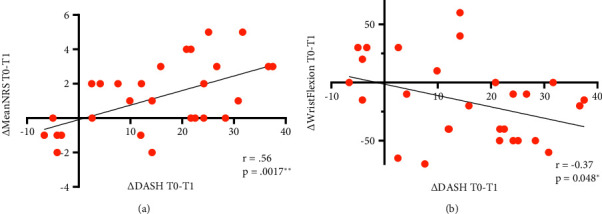
DASH as a CRPS disease monitor: correlation with pain and wrist flexion. Differences (∆) between the time point of inclusion (T0) and follow-up (ø 2.5 years later, T1) were calculated. (a) Relationship of ∆mean NRS and ∆DASH. Positive values indicate improvement of function or pain. (b) Association of ∆DASH with ∆wrist flexion. A negative ∆wrist flexion indicates a better movement after ø 2.5 years, e.g., 30° at T0 and 40° at T1. ^*∗*^*p* < 0.05. ^*∗∗*^*p* < 0.01. Pearson's correlation, normal distribution.

Furthermore, we sought to ascertain whether the alteration in mean pain intensity correlated with the change in DASH, as we identified pain as a predictor for arm function (see section below). Indeed, these two parameters correlated strongly (Figure 3).

### 3.4. Apart from Pain, Only Age Predicts DASH

In order to identify potential predictors of high DASH scores, we conducted a multiple regression analysis, including twelve factors: CRPS type I or II, age, gender, depression and anxiety in the medical history, BMI, mean pain in the week before presentation at study centre, CSS, NPSI, von Korff, BDI, STAI-T. The *R*^2^ for DASH was 0.62 (adjusted *R*^2^ = 0.59), indicative for a high goodness of fit [33]. Higher levels in all scores representing pain (mean pain intensity, NPSI, and von Korff) and age predicted higher DASH values (Table 2).

### 3.5. Recovery of Active Wrist Flexion Is Associated with DASH Recuperation

The majority of patients with CRPS displayed severe restriction of active range of motion (ROM) in the wrist and the fingers D II and D V. A substantial number of subjects manifested a restricted wrist and D V flexion and all CRPS patients could not fully bend their index finger. Following a period of 2.5 years, significant improvements were observed in wrist and D II flexion, with an average increase of 20%. Conversely, no change was noted in D V flexion. Similarly, 80% continued to experience difficulties with wrist extension; there was a mere 10% improvement. Finger extension was restricted in ¼ of the CRPS cohort and did not improve over time (Table 3).

To understand if an improvement in DASH (ΔDASH T0-T1 positive) correlates with an enhancement in active ROM of the wrist, we calculated the Person's correlation coefficient. A positive ΔROM wrist reflects a reduced angle; a negative ΔROM reflects a larger angle and thereby an improvement of function. We found a moderate negative correlation, meaning better ROM is associated with better DASH (Figure 3(b)). No other correlations between ΔDASH and ΔROM wrist extension, D II and D V flexion, and extension were significant. About two-thirds of patients improved in DASH and wrist flexion, 55% in DASH and D II flexion, as well as 40% in DASH and D V flexion. Less changes were observed in the improvement of wrist extension: 45% of patients enhance in DASH and wrist extension and about 20% in DASH and D II and D V (Table 3).

## 4. Discussion

The principal findings of the present study are that CRPS, especially CRPS type II, of the upper limb had a comparable or even greater impact on daily functioning than brachial plexus avulsions, and that the impairment of daily functioning exhibited a slight, approximately 10%, improvement over time, even after guideline-compliant therapy [4, 21, 34]. Drivers for this upper limb impairment were pain, age, and restriction of the range of motion of hand joints.

The DASH score for CRPS was comparable to that observed in plexus lesion patients. Thus, the limitations of daily living are equivalent to those seen in a paralyzed and painful arm after a brachial plexus injury, with no prospect of recovery. The DASH sores of our patients are in the same magnitude as the scores in previous small studies and case reports [16, 35–37]. Motor dysfunction (reduced strength), numbness, and reduced range of motion, 80% in our group, pose major challenges for CRPS patients and critically affect the return to work [6, 7, 38–40]. This could be addressed by disease-specific rehabilitative therapy programs [34].

DASH subitems requiring muscle power from several muscle groups of the upper extremity like sports such as golf or tennis (question 18), opening a tight jar (question 1), and gardening (question 8) were mostly affected in CRPS patients. Less affected were activities that are more independent of hand function such as sexual activity (question 21) or requiring less but better self-controlled muscle power such as handwriting (question 2) or turning a key (question 3). Interestingly, the items most likely to improve are those that are also known to cause the highest loss of quality of life and ability to work, such as question 18, weakness in arm, shoulder, and hand (question 27), and activity-related pain (question 25). Identification of the most important impairments could lead to the development of personalized treatment addressing specifically these particular deficits in future studies.

What could be the reasons for the high DASH score in CRPS? Impaired motor function and pain could be the result of changed cortical representation including sensory-motor integration of the affected hand [41]. Sensory motor retraining with mirror therapy or graded motor imagery may be able to partly reverse this process [42]. In rare cases, simple motor impairment and pain-related weakness even progress to dystonia, which is characterized by involuntary muscle contractions significantly limiting voluntary arm and hand use, or myoclonus [43].

We found a strong positive indication that change in pain changes the DASH report during the CRPS course. This means that pain is a major driver of loss of limb function. Savaş et al. and Bean et al. also identified severe pain as a trigger of disability as well as numbness of the hand [16, 38]. Multiple regression analysis isolated age in addition to pain as another risk factor for reduced arm function and pain-related disability (von Korff score). We found no previous investigations on the impact of age on CRPS recovery; however, age is associated with poorer outcomes after distal radius fractures [44]. Subsequent studies should validate this specifically for CRPS. Depression and anxiety as reported by clinical questionnaires (BDI-II and STAI-T) were of low prevalence in our CRPS cohort. Although anxiety and depression are important comorbidities in chronic pain [45, 46], neither was a predictor of the DASH score or reduced arm function. However, the fear-avoidance model provides a rationale for movement avoidance in CRPS [47, 48]. Thus, our results differ from previous studies, where lower anxiety was associated with better outcomes related to pain and disability [49, 50]. In contrast to pain, psychological comorbidities seem to play a less important role for limb function in CRPS. Our study has some limitations. This first is the different sizes of the groups due to the rarity of plexus lesions. Second, this study is a retrospective chart analysis which has to rely on documented measurements. However, the ncRNAPain cohort was sampled with strict standard operating procedures so that documentation of symptoms should be complete. Third, treatment during follow-up was not entirely standardized. According to the German CRPS guideline, treatment could either comprise an outpatient treatment program, which emphasized the administration of pain medication and the prescription of occupational and physiotherapy, or an inpatient day clinic multidisciplinary program, which spanned 4–8 weeks. However, high-quality research demonstrating treatment evidence is still lacking [4].

To summarize, this present study proved that upper limb CRPS motor function should be much more in the focus of research and treatment. Reasons why this has not been done in the past may include a lack of suitable and standardized assessments. The DASH score could fill the gap for assessment and therapy monitoring.

## 5. Conclusion

CRPS is associated with a high degree of impairment of the affected extremity in this study in everyday life, comparable to the disability of patients with brachial plexus lesions. The conditions hardly improved in the course of time, not by adequate therapy either. Pain, age, and restriction in the range of motion of hand joints contributed to these findings. To assess the daily function, we used the 30-item DASH questionnaire. To facilitate its use in clinical trials, the development of a CRPS-specific DASH would be beneficial and should be considered for further investigations. The existence of pain and reduced joint mobility limiting the daily function of an extremity has already been described in the literature. The influence of age should be studied in future trials.

## Figures and Tables

**Table 1:** Epidemiological data.

Variables	CRPS upper limb (*N* = 193) Type I/II 167 (86.5%)/26 (13.5%)	Follow-up (*N* = 33)	Fracture controls (*N* = 36)	Plexus lesions (*N* = 12)
Gender (%) female/male	148 (86.5)/45 (23.3)	28 (75.8)/8 (24.2)	18 (50)/18 (50)	2 (17)/10 (83)
Age mean (range) (S.D.)	51.7 (18–77) (±12.5)	53.2^#^ (22–73) (±10.3)	47.9 (20–78) (±14.1)	33.9 (±12.4)
Body mass index mean (range) (S.D.)	27 (16–46) (±5.5)	26.4 (19.8–40.5) (±4.9)	25 (16–36) (±4.2)	—
DASH score mean (range) (S.D.)	54.7 (5.83–94.17) (±21)	41.3 (0.8–89.2) (±25.2)	21.2 (0–85.3) (±21.4)	51.4 (19.2–85) (±15.3)
CSS score mean (range) (S.D.)^†^^*∗∗∗*^	10.9 (4–17) (±2)	6.4 (0–16) (±3.4)	1.5 (0–9) (±2)	—
von Korff score mean (range) (S.D.)^†^^*∗∗*^	3 (0–4) (±1.2)	2.1 (0–4) (±1.2)	1.3 (0–4) (±1.3)	—
NPSI score mean (range) (S.D.)^†^	36.2 (0–100) (±23.8)	40.1 (9–93) (±23.9)	7.9 (0–43) (±10.4)	29.6 (5–83) (±20.6)
Mean pain mean (range) (S.D.)^†^^*∗∗*^	4.9 (0–10) (±2.3)	3.8 (0–8) (±2.3)	1.6 (0–7) (±2)	4.6 (1–8) (±2.3)
Current pain mean (range) (S.D.)^†^^*∗*^	4.1 (0–10) (±2.5)	3.2 (0–8) (±2.5)	0.9 (0–10) (±2.1)	4.5 (0–7) (±2.2)
Maximum pain mean (range) (S.D.)^†^^*∗*^	7 (0–10) (±2.4)	6.2 (0–10) (±3.1)	2.9 (0–10) (±3.3)	7.3 (4–10) (±1.7)
BDI-II score mean (range) (S.D.)	13.1 (0–54) (±3.1)	14 (0–47) (±13)	3.4 (0–12) (±3.4)	—
STAI-T score mean (range) (S.D.)	41 (0–76) (±14.8)	44.5 (34–58) (±5.7)	33.2 (22–46) (±6.2)	—
Depression in the medical history (%) yes/no/missing	54 (28)/137 (71)/2 (1)	10 (30.3)/23 (69.7)/0	3 (8.3)/33 (91.7)/0	—
Anxiety in the medical history (%) yes/no/missing	18 (9.3)/174 (90.2)/1 (0.5)	4 (12.1)/29 (87.9)/0	1 (2.8)/35 (97.2)/0	—
Pain duration in years mean (range) (S.D.)	1.3 (0–15.8) (±2)	2.5 (±3.6)	0.4 (0–1.7) (±0.5)	—

CRPS = complex regional pain syndrome group; follow-up group after ø 2.5 years *N* = 33; fracture controls *N* = 36: subjects with normal trauma healing; plexus lesions *N* = 12: patients after traumatic dorsal root avulsion. #at study inclusion. †Comparison between 33 out of the 193 CRPS patients at study inclusion with the same patients as 33 follow-ups after ø 2.5 years with significance ^*∗*^*p* < 0.05, ^*∗∗*^*p* < 0.01, and ^*∗∗∗*^*p* < 0.001, chi-squared test, Mann–Whitney *U* test as appropriate for not normally distributed data.

**Table 2:** Probable risk factors for higher DASH scores: pain and age.

Variable	DASH score		
	Not standardized	Std. err	Standardized
Constant	−27.402	16.283	
CRPS type	6.080	3.322	0.169
Age	0.356^*∗∗∗*^	0.093	0.204
Gender	−4.810	2.828	−0.094
Depression	5.831	2.946	0.126
Anxiety	−2.913	4.047	−0.041
Body mass index	0.219	0.212	0.054
Mean pain	1.876^*∗∗*^	0.644	0.200
CSS score	0.289	0.405	0.040
NPSI	25.776^*∗∗∗*^	6.015	0.288
von Korff score	5.470^*∗∗∗*^	1.165	0.301
BDI-II score	0.113	0.154	0.061
STAI-T score	0.268^*∗*^	0.130	0.099
*R* ^2^	0.616		
Corr. *R*^2^	0.586		
*F* (df = 12; 156)	20.837		

Multiple linear regression for CRPS patients at first contact. Coefficients (standardized and not standardized) and standard error (Std. err.) of constants and variables. Coefficient of determination (*R*^2^), corrected coefficient of determination (corr. *R*^2^), and F-statistics. Significance ^*∗*^*p* < 0.05, ^*∗∗*^*p* < 0.01, and ^*∗∗∗*^*p* < 0.001.

**Table 3:** Improvement of hand function in CRPS patients in the follow-up.

Joint movement (%)	T0 pathologic	T1 pathologic	ROM: ∆T (T0-T1) improvement	DASH and ROM improvement
Wrist extension	85.0	70.0	48.5	44.8
Wrist flexion	79.0	64.0	66.7	62.0
Finger D2 flexion	100.0	81.2	63.6	55.2
Finger D5 flexion	84.8	81.8	48.5	41.4

Joint movement at the hand was measured using a goniometer at maximal active flexion and extension of indicated joints. Normal range of motion (ROM) was defined as wrist extension/flexion of 60/70°, D2 flexion of 110°, and D5 flexion of 100°. Pathologic movement was specified as 10° less than normal. Since the finger extension hardly showed any conspicuous features, we refrained from listing it here. The percentage of CRPS patients was calculated (*N* = 33).

## Data Availability

The data are available on request from the corresponding author and are not available publicly due to restrictions to the privacy of research participants.
